# Codesign of Mental Health Interventions With Young People From Racially Minoritised Populations: A Systematic Review of Methods and Outcomes

**DOI:** 10.1111/hex.70204

**Published:** 2025-03-06

**Authors:** Ishrat Shahnaz, Kelly Rose‐Clarke, Daniel Michelson, Petra C. Gronholm

**Affiliations:** ^1^ Department of Child and Adolescent Psychiatry Institute of Psychiatry, Psychology & Neuroscience, King's College London London UK; ^2^ Department of Psychology University of Dhaka Dhaka Bangladesh; ^3^ UCL Institute for Global Health University College London London UK; ^4^ NIHR Maudsley Biomedical Research Centre South London and Maudsley NHS Foundation Trust and King's College London London UK; ^5^ Centre for Global Mental Health and Centre for Implementation Science, Health Service and Population Research Department Institute of Psychiatry, Psychology & Neuroscience, King's College London London UK; ^6^ Centre for Global Mental Health, Department of Population Health London School of Hygiene & Tropical Medicine London UK

**Keywords:** codesign, mental health intervention, racially minoritised groups, young people

## Abstract

**Background:**

Codesign of mental health interventions entails the active involvement of end users and other stakeholders in various stages of the developmental process. This has emerged as a promising approach for developing evidence‐based mental health interventions aligned with minoritised populations' needs and preferences. However, key questions remain about the methods and outcomes of codesign studies focused on young people from racially minoritised groups. The current review aimed to explore the codesign approaches and phases used in developing mental health interventions with young people from racially minoritised populations, analyse the codesign outcomes for participants and examine the contextual enablers and barriers impacting the codesign process.

**Methods:**

A systematic search was conducted across MEDLINE, EMBASE, PsycINFO, Global Health, Web of Science and Scopus. Citations and references of included studies were also checked. Study quality and reporting of codesign were assessed using the Mixed Method Appraisal Tools and the Guidance for Reporting Involvement of Patients and the Public‐2 checklist. Data were synthesised using narrative synthesis, content analysis and meta‐synthesis.

**Results:**

Eighteen eligible studies reported various codesign and participatory approaches, including community‐based participatory research, co‐production, human‐centred design, youth and family codesign model, community engagement research, community development model, participatory evaluation model, participatory research design approach and community participatory research partnership. The most common codesign stages followed were exploring problems and solutions, ideating and creating, and refining. In terms of outcomes, the reported benefits of codesign for young people included personal development and well‐being, enhanced knowledge and career skills, and better mental health outcomes. Codesigning with youth and other stakeholders (e.g., family members, other caregivers, community members and practitioners) also improved the research projects by identifying specific problems, increasing participant recruitment and enhancing data collection. Additionally, other stakeholders gained a platform to share their expertise, understand youth mental health and build capacity through codesign. Regarding enablers and barriers, reducing power differentials, fostering community engagement and collaboration with other stakeholders facilitated the codesign process, whereas barriers included lack of resources, power imbalances, lack of rapport building and selection bias.

**Conclusions:**

This review outlines the potential benefits of codesign for developing mental health interventions for racially minoritised youth. These benefits include continuous stakeholder engagement to understand community needs better, reducing power differentials and building trust through culturally tailored activities and communication strategies.

**Patient and Public Contribution:**

Patients and the public did not contribute directly to this review though the reviewed literature was specifically concerned with participatory research activities.

## 1 Background

The term ‘racially minoritised’, coined by Gunaratnum (2003), recognises that minoritisation is an active process, whereby ‘people are actively minoritised by others rather than naturally existing as a minority, as the terms racial minorities or ethnic minorities imply’ [1]. Racially minoritised groups often face disparities in mental health care access, experiences and outcomes [2, 3]. Young people from these backgrounds are less likely to initiate care and more likely to discontinue treatment prematurely [4, 5]. Despite progress in research, practice and policy, many of these children and young people still experience poor mental health outcomes and have lower access to mental healthcare services [6]. These inequalities are largely attributed to systemic racial bias within healthcare systems [7, 8]. Additionally, racially minoritised families prefer primary care, perceiving it as less stigmatising than specialised mental health services. However, this preference can be challenging when primary care options are not readily available [4, 8, 9].

The poorer outcomes and engagement seen in minoritised groups can also be attributed to a lack of culturally competent care or care that does not align with evidence‐based guidelines. Many interventions are based on Western concepts of mental disorder [10], which may not align with diverse cultural perceptions of mental health [11] or broader cultural diversity [12]. Research shows that culturally adapted interventions are more effective than non‐adapted ones for racially minoritised people [13, 14, 15], as culture shapes beliefs and understandings of mental health [10]. These adaptations enhance the effectiveness, accessibility and acceptability of treatments to individuals with different cultural needs [16, 17], while fostering trust and respect, crucial for a strong therapeutic relationship [17].

To improve mental health outcomes, involving end users and other stakeholders from minoritised groups in research is important. Participatory approaches—a broad category of methods that engage people in the design process—have proven effective, with codesign being a particularly impactful approach. Codesign is a specific form of participatory research that focuses on making decisions in partnership with people who will use, implement and/or otherwise engage with mental health services and interventions [18]. It is a shift away from the traditional design of services and interventions by clinicians and academics ‘for’ users, towards a more collaborative approach of designing ‘with’ users [19]. Codesign values lived experience as essential expertise and draws on principles from participatory design research to ensure that potential end users are active design decision makers throughout the design process [19, 20, 21]. Since racially minoritised youth experience barriers to accessing mainstream mental health services [22], involving them in the codesign process allows the other stakeholders to understand the challenges better and tailor interventions accordingly [23].

Notwithstanding these potential benefits, codesign presents several complexities. Blomkamp [24] warns that poorly designed, inadequately facilitated or manipulative participatory projects can heighten distrust among minoritised participants towards key project partners. Mark and Hagen [25] also observed that people are wary of the term ‘codesign’, experiencing ‘codesign fatigue’ due to its inconsistent application and lack of genuine power sharing, participation or partnership. They believe the term has lost its true meaning. Other issues related to the use of codesign, specifically with racially minoritised young people, include challenges in the inclusion of diverse expertise in a safe environment, managing power imbalances and addressing the risk of tokenism, which may prevent their expertise from being genuinely heard and valued [26]. Ensuring that these expertise are heard and valued builds trust and enhances the relevance, utilisation and overall effectiveness of the intervention [27].

To the best of the authors' knowledge, this is the first systematic review of the available literature on codesigning mental health interventions with racially minoritised youth aged 10–24 (in line with the World Health Organization's definition of ‘young people’ [28]). No previous reviews have provided an overview of the current practice of codesign with this group, its impact on outcomes or its approach to addressing factors that support or impede codesign. This study aims to close this gap by addressing the following research questions (RQs):

**RQ1.** *What codesign approaches have been used to develop mental health interventions with young people from racially minoritised populations?*

**RQ2.** *At what phase(s) of the developmental process have codesign approaches been used, and how does codesign influence the outputs of each phase?*

**RQ3.** *What are the outcomes of codesign for the individual participants?*

**RQ4.** *What contextual enablers and barriers influence the process and outcomes of codesign for different stakeholder groups?*

## 2 Materials and Methods

This systematic review protocol was pre‐registered with PROSPERO (CRD42023443117). The review has been reported following the Preferred Reporting Items for Systematic Reviews and Meta‐Analyses (PRISMA) guidelines [29]. Please see Supporting Information S1 for PRISMA checklist.

### 2.1 Search Strategy

Peer‐reviewed literature was systematically searched from 16 to 21 July 2023 on the following databases: MEDLINE, EMBASE, PsycINFO, Global Health, Web of Science and Scopus. Only studies published in English were considered, without any publishing date restrictions. The first author (I.S.) conducted the searches in each database using key search terms and subject headings related to ‘codesign,’ ‘mental health’, ‘intervention’, ‘young people’ and ‘racially minoritised groups’ (see Supporting Information S2 for full search strategy). Search strings were adjusted for each database. We also checked the reference and citation lists of included studies to identify additional eligible studies.

### 2.2 Eligibility Criteria and Selection of Studies

#### 2.2.1 Participants/Populations

Eligible studies described the codesign of mental health interventions, where the intended end users were principally young people from racially minoritised groups. Codesign participants could be prospective end users or relevant stakeholders including family members, other caregivers, community members and practitioners. We also included studies where ‘young people’ might span a wider age range than 10–24 years, provided that the mean age of the youth participants fell within the targeted age range.

#### 2.2.2 Codesign Condition

**Figure 1:**
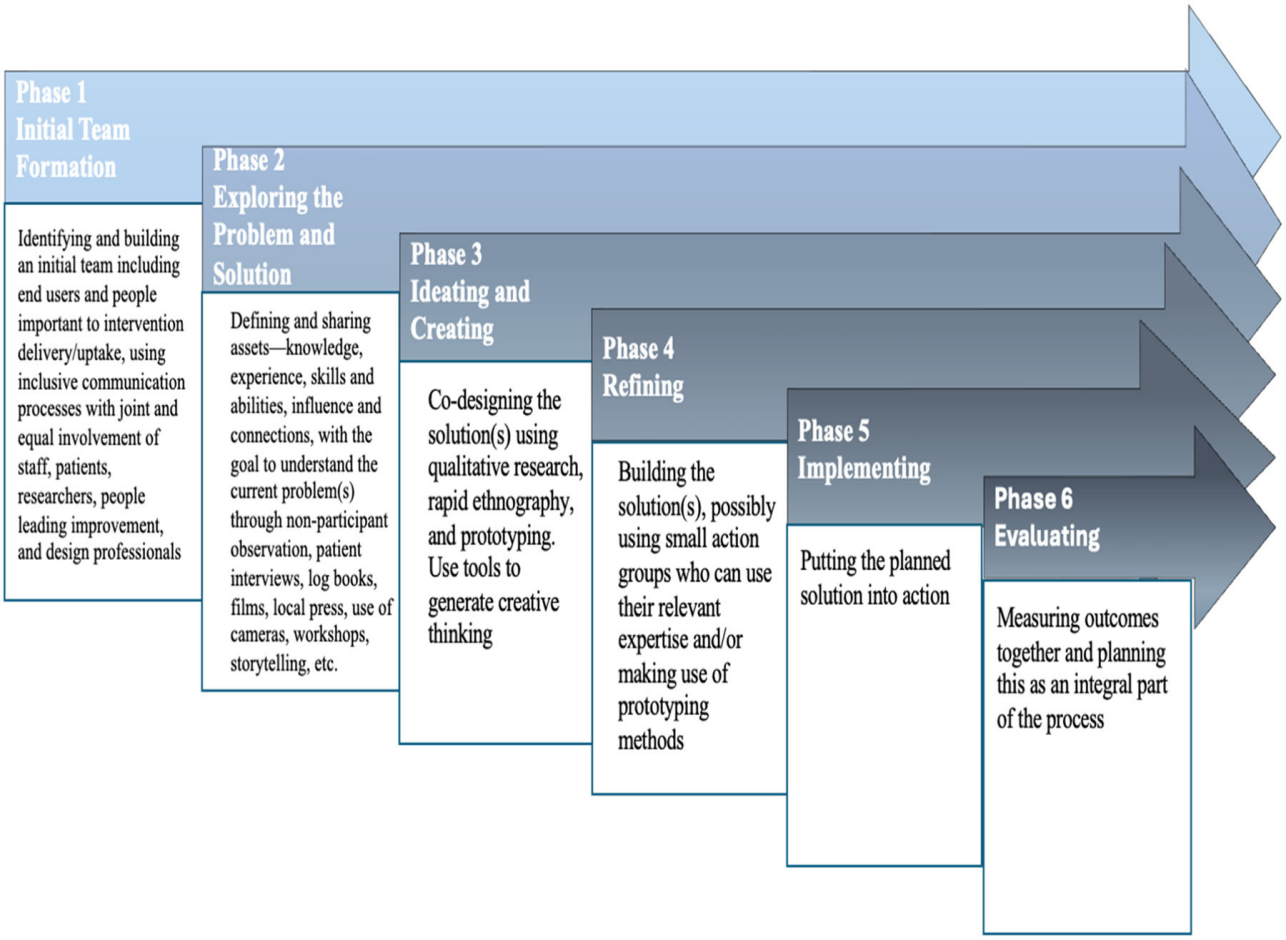
Interventions development phases in which codesign approaches were utilised, based on the typology of O'Cathain et al. [18].

A study was considered to involve codesign if the people for whom the intervention aims to help, [we]re involved in decision‐making about the intervention throughout the development process [18]. For a study to be eligible, we required this involvement in at least two steps of the developmental process using a typology developed by O'Cathain et al. [18] (see Figure 1).

#### 2.2.3 Interventions

We included studies where codesign activities centred on interventions to prevent or treat common mental health problems (i.e., depression, anxiety, conduct difficulties, trauma, post‐traumatic stress disorder [PTSD], obsessive‐compulsive disorder [OCD] and/or global psychopathology), as well as studies concerned with codesigning mental health promotion programmes (i.e., where the aim was to promote positive mental health). Relevant interventions were universal, indicated or selective [30], incorporating specific behavioural, cognitive, interpersonal and/or emotion‐focused elements [31]. Interventions were delivered by any mode (e.g., in‐person, digital/web‐based). We included studies where the intended intervention involved direct sessions with young people and/or joint or parallel sessions with parents, caregivers and teachers as long as young people were involved somewhere in the intervention process.

#### 2.2.4 Outcomes

Studies were included if they reported on processes and outcomes related to codesign. Specifically, we included studies that described codesign activities and their influences across different phases of intervention development, the impact of codesign practices on the stakeholders, and the barriers and facilitators to codesign.

#### 2.2.5 Study Design

The review included studies of any design (quantitative, qualitative and mixed‐method) if they described the use of codesign in the adaptation or development of the intervention.

### 2.3 Data Screening and Extraction

The citations were uploaded to EndNote and duplicates were removed. I.S. screened the title and abstract of each report, and 10% (3012 records) were cross‐checked by another reviewer independently against the inclusion and exclusion criteria. Disagreements were resolved through discussion between raters. Interrater reliability was substantial (Cohen's kappa = 0.67) [32]. Ineligible studies were removed, and potentially eligible studies were retained for full‐text review. I.S. completed full‐text screening, and a further proportion (35%; 47 studies) was checked independently by another reviewer, with substantial agreement (Cohen's kappa = 0.75) [32]. Any disagreements were resolved through discussion with senior authors. Data were extracted from all eligible papers with the aid of a structured proforma, including information about the lead author, year, location, study design, codesign participants, codesign framework/model used, codesign steps and activities, codesign outcomes, intervention details, intended end users, delivery method and primary outcome of the intervention.

### 2.4 Quality Assessment

The eligible articles were assessed for quality (i.e., risk of bias) using the Mixed Methods Appraisal Tool (MMAT) [33]. Qualitative and quantitative studies were evaluated based on five criteria while mixed‐method studies were assessed using 15 criteria. The criteria were rated as ‘yes’ (criterion is met), ‘no’ (criterion is not met) and ‘cannot tell’ (not enough information to judge). The total MMAT score was converted into an overall percentage, with 0% indicating the lowest quality and 100% indicating the highest quality. The methodological quality of each study was assessed independently by I.S. and 25% by another reviewer, with substantial agreement (Cohen's kappa = 0.75). No study was excluded from the review based on their MMAT assessment [33].

Quality of reporting of codesign was assessed using the GRIPP 2 short form (Guidance for Reporting Involvement of Patients and the Public) [34]. I.S. rated each study independently using the scoring strategy where a rating of ‘yes’ (sufficient information = one star ★), ‘limited’ (not sufficient information to judge = half star ☆) and ‘no’ (no information = no star) were assigned based on the sufficiency of reporting in the original study. These ratings contributed to an overall star rating where the total score could range from zero to a maximum of five stars. Despite not being a quality checklist per se, GRIPP 2 aims to provide useful information about the adequacy of reporting for participatory approaches such as codesign [35].

### 2.5 Data Synthesis

We employed narrative synthesis, content analysis and meta‐synthesis to integrate key findings from the included studies and tabulated study characteristics to summarise findings. Specific synthesis methods for each research question are detailed below.

#### 2.5.1 RQ1: What Codesign Approaches Have Been Used to Develop Mental Health Interventions With Young People From Racially Minoritised Populations?

Where available, we extracted information on the specific codesign framework or model used in each study. We summarised these results narratively.

#### 2.5.2 RQ2: At What Phase(s) of the Developmental Process Have Codesign Approaches Been Used, and How Does Codesign Influence the Outputs of Each Phase?

We conducted a content analysis to categorise and code the stages of codesign research using the typology in Figure 1. We also categorised the data collection methods implemented at each stage (e.g., focus group discussions, interviews, ranking and mapping). Narrative summaries were then provided for each category to synthesise and interpret the findings.

#### 2.5.3 RQ3: What Are the Outcomes of Codesign for the Individual Participants?

We employed content analysis to categorise the reported benefits of codesign for participating young people, the research team and their project, and the other stakeholders (e.g., family members, other caregivers, community members and practitioners). Specifically, we considered benefits to young people in terms of advantages gained through their involvement in codesign activities; benefits to the research project in terms of how the research team accrues depth of knowledge and experiences to inform, guide and produce more meaningful research aims, methods and outcomes, and benefits to the other stakeholders who participated themselves or were otherwise involved in terms of the impact of codesign on their collaboration, partnerships and community networks. We narratively summarised the findings to provide a contextualised account of these outcomes.

#### 2.5.4 RQ4: What Contextual Enablers and Barriers Influence the Process and Outcomes of Codesign for Different Stakeholder Groups?

We followed an established guideline for meta‐synthesis [36]. Relevant textual data on contextual enablers and barriers from the Results and Discussion sections of the included articles were extracted verbatim and recorded in a table for coding. Through a deductive‐inductive process, data were coded thematically and compared with each other to find patterns of similarities and differences. Related themes were identified, grouped and compared, resulting in central categories of enablers and barriers, which were then elaborated narratively.

## 3 Results

**Figure 2:**
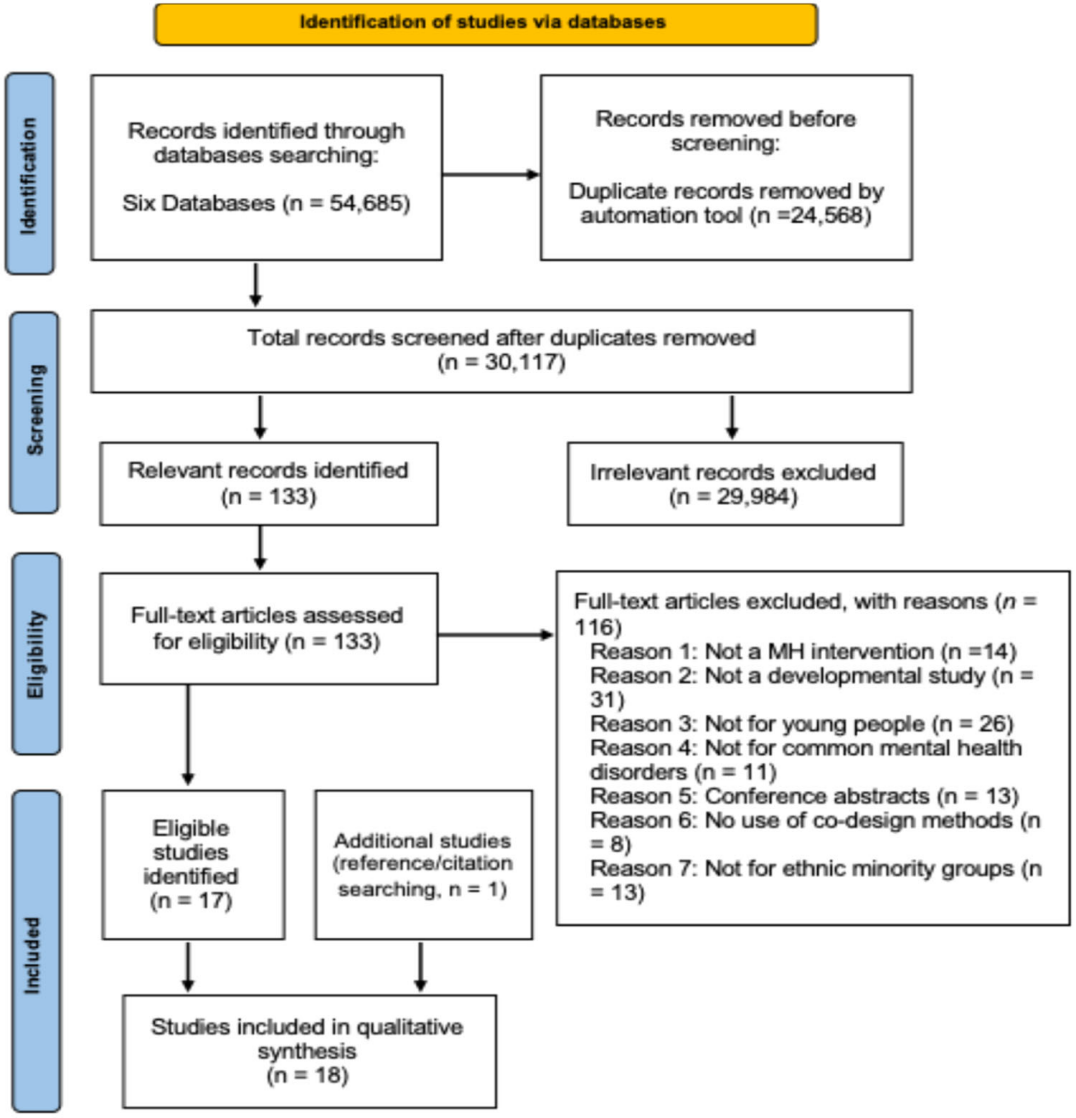
The Preferred Reporting Items for Systematic Reviews and Meta‐Analyses flow diagram.

The PRISMA flow diagram (Figure 2) summarises the process of identifying and selecting studies for inclusion. Out of 54,685 records, 30,117 papers were screened based on title and abstract after removing duplicates, resulting in 133 full‐text studies for consideration. After applying eligibility criteria, 116 studies were excluded, leaving 17 eligible peer‐reviewed studies. Manual searching of references and citation lists yielded one additional study, bringing the total number of included peer‐reviewed studies to 18.

### 3.1 Study Characteristics

Table 1 summarises the characteristics of the included studies, conducted between 2002 and 2023 across six countries, primarily the United States (*n* = 9; 50%). Other studies were conducted in Lebanon (*n* = 2; 11%), Australia (*n* = 2; 11%), Canada (*n* = 2; 11%), Tanzania (*n* = 1; 6%) and the United Kingdom (*n* = 2; 11%). Twelve (67%) studies used qualitative approaches, with six (33%) using mixed‐method. Ten studies (56%) focused on developing or adapting interventions in community settings, including refugee camps (*n* = 2; 11%), education centres (*n* = 1; 6%), hospitals (*n* = 2; 11%), service centres (*n* = 1; 6%), health centres (*n* = 1; 6%) and other community venues (*n* = 3; 17%). Five studies (28%) took place within schools, whereas three (17%) spanned both school and community settings. Twelve studies (67%) included youth alongside carers, family members, practitioners and/or other community members in codesign activities. Four studies (22%) included only carers, family members, practitioners and other community members, whereas remaining two (11%) involved young people exclusively. Although young people were the primary end users of all interventions and mental health programmes, eight studies (44%) included joint or parallel sessions with caregivers, parents and/or teachers. Fifteen studies (83%) targeted selective or indicated prevention interventions for at‐risk youth. Three studies (17%) developed mental health promotion programmes for minoritised youth across their communities, regardless of individual risk.

**Table 1:** Characteristics of included studies (*n* = 18).

Lead author, year	Location (country)	Study design	Intervention delivery settings	Intervention	Intervention type	Intended end user				Primary intended outcome(s) of the intervention
						End user	Age	Ethnicity	Mental health condition	
Afifi et al., 2011	Lebanon	Mixed‐method	Refugee camp	Qaderoon (Arabic for ‘we are capable’)	Promotion	1. Youth 2. Parents 3. Teachers	1. 10–14 years	Palestinian	1. Youth population within the camp	Improve mental well‐being
Bond et al., 2007	Australia	Qualitative	Community education centres, hospitals	The Changing Cultures Project	Promotion	1. Youth	1. 16–24 years	Asian, Eastern European, African, Middle Eastern, and other	1. All refugee and newly arrived young people within the community	Enhance positive mental health and well‐being
Brown et al., 2020	Lebanon	Qualitative	Community‐based settings (not specified)	Early Adolescent Skills for Emotions (EASE)	Selective prevention	1. Adolescents 2. Caregivers	1. 10–14 years	Syrian, Lebanese, and Palestinian	1. Living in adversity and at risk of emotional distress	Reduce psychological distress
Clarke et al., 2022	United States	Mixed‐method	After‐school programme	Resilient In spite of Stressful Events (RISE)	Indicated prevention	1. Adolescents	NR	Black	1. At increased risk of developing mental health problems	Reduce depressive symptoms and aggression
El Guenuni et al., 2022	United Kingdom	Mixed‐method	Hospital and community centre	A Therapeutic group	Indicated prevention	1. Youth	1. 11–14 years	Moroccan	1. Affected by collective trauma and loss	Prevent the escalation of mental health issues and increase access to mental health services
Etter et al., 2019	Canada	Qualitative	Schools, community centres	Youth mental health services transformation project: ACCESS Open Minds (ACCESS OM)	Promotion	1. Youth	NR	Inuit	1. All youth in the community regardless of individual risk level	Increase overall mental health literacy and wellness
Fabian et al., 2023	United States	Qualitative	Not explicitly reported	Sustainable Technology for Adolescents to Reduce Stress (STARS)	Selective prevention	1. Youth and young adults	1. 14–25 years, mean age 17.7	NR	1. At risk of developing common mental disorders	Reduce symptoms of common mental disorders
Henderson et al., 2023	Canada	Qualitative	Community service centres	Youth Wellness Hubs Ontario (YWHO)	Selective prevention	1. Adolescents and young adults	1. 12–25 years, mean age 18.5	Linguistically and culturally diverse communities	1. No risk factor/clinical group	Enhance early intervention services addressing mental health, substance use, primary care and other social needs
Kataoka et al., 2006	United States	Qualitative	Schools	Cognitive Behavioral Intervention for Trauma in Schools (CBITS)	Indicated prevention	1. Youth 2. Parents 3. Teachers	NR	Latino immigrant	1. Trauma‐related symptoms from violence exposure	Reduce symptoms of PTSD, depression and general anxiety
King and Said, 2019	United Kingdom	Mixed‐method	NHS child and adolescent mental health service	A psychological skills group	Selective prevention	1. Young people	1. Mean age 16.36	Unaccompanied asylum‐seeking and refugee (e.g., Afghanistan, Ethiopia and Sudan)	1. At risk of psychological distress	Increase mental well‐being
Mance et al., 2010	United States	Qualitative	Youth opportunity programme centre	Structured Psychotherapy for Adolescents Responding to Chronic Stress (SPARCS)	Indicated prevention	1. Adolescents and young adults	1. 16–24 years	African American	1. Chronically exposed to trauma or severe stress	Reduce the worsening of depressive symptoms and increase adaptive coping strategies
Mulvaney‐Day et al., 2006	United States	Qualitative	Schools	Systems intervention (a series of interventions in a public school system)	Selective prevention	1. Young	NR	African American, Hispanic, Asian, Native American, White and other	1. At risk of developing disruptive behaviour problems	Improve behavioural and academic functioning
Povey et al., 2020	Australia	Mixed‐method	Schools, drug rehabilitation service	Aboriginal and Islander Mental Health Initiative for Youth (AIMhi‐Y) App	Indicated prevention	1. Youth	1. 10–18 years	Aboriginal and Torres Strait Islander	1. Experiencing psychological distress	Reduce psychological distress and the risk of suicide
Reaven et al., 2020	United States	Qualitative	Schools	Facing Your Fears (FYF)	Indicated prevention	1. Young people 2. Parents	1. 8–14 years, mean age 11	Hispanic/Latinx; African American	1. ASD and anxiety	Reduce anxiety and emotion dysregulation
Saulsberry et al., 2013	United States	Qualitative	Community health centres	Chicago Urban Resiliency Building (CURB)	Indicated prevention	1. Adolescents 2. Parents	1. 13–17 years	African‐ American and Latino	1. Elevated risk of depressive disorders	Prevent depression
Shelton et al., 2005	United States	Qualitative	Not explicitly reported	Leadership, Education, Achievement, and Development (LEAD) programme	Selective prevention	1. Adolescents 2. Parents	1. 10–14 years	African American	1. At risk of a combination of factors such as poverty, school difficulties, youth disruptive behaviours and family disruptions	Reduce the risk of minority youth involvement in the juvenile justice system
Singh et al., 2021	Tanzania	Mixed‐method	Refugee camp	EASE	Indicated prevention	1. Adolescents 2. Caregivers	10–14 years	Burundian	1. Exhibiting internalising problems (e.g., symptoms of depression, anxiety or distress)	Improve mental health
Stein et al., 2002	United States	Qualitative	Schools	Mental Health for Immigrants Program (MHIP)	Indicated prevention	1. Children 2. Parents 3. Teachers	NR	Immigrant	1. Symptoms of psychological distress	Reduce symptoms of PTSD and depression

### 3.2 Result of the Quality Assessment

Overall, the MMAT ratings suggested high quality among the studies (see Table 2). Of the twelve qualitative studies, ten met all five quality criteria. Two studies met only two criteria raising concerns about their data collection methods, interpretation of results and coherence between data sources, collection, analysis and interpretation. Among the six mixed‐method studies, only two met all 15 criteria. The remaining four fell short on four to five criteria, reflecting issues with confounders, integration between quantitative and qualitative results and adherence of the study components to the quality criteria of each method involved.

**Table 2:** Quality of studies based on MMAT criteria.

First author, year	1. Qualitative design					2. Quantitative (randomised) design					3. Quantitative (descriptive) design					4. Mixed‐methods design					MMAT total score (%)
	1.1	1.2	1.3	1.4	1.5	2.1	2.2	2.3	2.4	2.5	3.1	3.2	3.3	3.4	3.5	4.1	4.2	4.3	4.4	4.5	
Afifi et al., 2011	Y	Y	Y	Y	Y						Y	Y	Y	?	Y	Y	Y	Y	Y	?	80
Bond et al., 2007	Y	Y	Y	Y	Y																100
Brown et al., 2020	Y	Y	Y	Y	Y																100
Clarke et al., 2022	Y	Y	Y	Y	Y	?	?	Y	?	Y						Y	Y	Y	?	?	40
El Guenuni et al., 2022	Y	Y	Y	Y	Y						Y	Y	Y	Y	Y	Y	Y	Y	Y	Y	100
Etter et al., 2019	Y	Y	Y	Y	Y																100
Fabian et al., 2023	Y	Y	Y	Y	Y																100
Henderson et al., 2023	Y	?	Y	?	?																40
Kataoka et al., 2006	Y	Y	Y	Y	Y																100
King and Said, 2019	Y	Y	Y	Y	Y						Y	Y	Y	Y	Y	Y	Y	Y	?	Y	80
Mance et al., 2010	Y	Y	Y	Y	Y																100
Mulvaney‐Day et al., 2006	Y	Y	Y	Y	Y																100
Povey et al., 2020	Y	Y	Y	Y	Y						Y	Y	Y	?	Y	Y	Y	Y	Y	Y	80
Reaven et al., 2020	Y	Y	Y	Y	Y																100
Saulsberry et al., 2013	Y	Y	Y	Y	Y																100
Shelton et al., 2005	Y	Y	Y	Y	Y																100
Singh et al., 2021	Y	Y	Y	Y	Y						Y	Y	Y	Y	Y	Y	Y	Y	Y	Y	100
Stein et al., 2002	Y	?	Y	?	N																40

*Note:* Mixed Methods Appraisal Tool (MMAT), version 2018 (Hong et al. [33]). (1) Qualitative domain questions: (1.1) Is the qualitative approach appropriate to answer the research question? (1.2) Are the qualitative data collection methods adequate to address the research question? (1.3) Are the findings adequately derived from the data? (1.4) Is the interpretation of results sufficiently substantiated by data? (1.5) Is there coherence between qualitative data sources, collection, analysis and interpretation? (2) Quantitative (randomised) domain questions: (2.1) Is randomisation appropriately performed? (2.2) Are the groups comparable at baseline? (2.3) Are there complete outcome data? (2.4) Are outcome assessors blinded to the intervention provided? (2.5) Did the participants adhere to the assigned intervention? (3) Quantitative (descriptive) domain question: (3.1) Is the sampling strategy relevant to address the research question? (3.2) Is the sample representative of the target population? (3.3) Are the measurements appropriate? (3.4) Is the risk of nonresponse bias low? (3.5) Is the statistical analysis appropriate to answer the research question? (4) Mixed‐methods domain questions: (4.1) Is there an adequate rationale for using a mixed‐methods design to address the research question? (4.2) Are the different components of the study effectively integrated to answer the research question? (4.3) Are the outputs of the integration of qualitative and quantitative components adequately interpreted? (4.4) Are divergences and inconsistencies between quantitative and qualitative results adequately addressed? (4.5) Do the different components of the study adhere to the quality criteria of each tradition of the methods involved?

Abbreviations: N = no, criterion is not met, Y = yes, criterion is met, ? = cannot tell, not enough information to judge.

The GRIPP 2 checklist ratings on the sufficiency of reporting codesign is summarised in Table 3. Only five studies (28%) met all criteria and thirteen (72%) met between one and four criteria. The included studies mostly reported the aim, methods and results of involvement activities, but with relatively little critical reflection about what went well and what did not.

**Table 3:** Sufficiency of reporting of the participatory approach by GRIPP 2 checklist.

First author, year	GRIPP 2 checklist					Sufficiency of reporting rating
	Involvement aim(s)	Involvement methods	Study results (outcomes of involvement)	Discussions and conclusions	Reflections/critical perspective	
Afifi et al., 2011	Y	Y	Y	Y	Y	★★★★★
Bond et al., 2007	L	Y	Y	N	Y	★★★☆
Brown et al., 2020	N	Y	N	N	L	★☆
Clarke et al., 2022	Y	L	L	N	N	★★
El Guenuni et al., 2022	Y	Y	Y	Y	Y	★★★★★
Etter et al., 2019	N	L	Y	Y	N	★★☆
Fabian et al., 2023	Y	Y	L	Y	Y	★★★★☆
Henderson et al., 2023	Y	N	N	N	N	★
Kataoka et al., 2006	Y	Y	Y	Y	Y	★★★★★
King and Said, 2019	N	L	L	N	L	★☆
Mance et al., 2010	Y	Y	Y	Y	Y	★★★★★
Mulvaney‐Day et al., 2006	Y	Y	Y	Y	Y	★★★★★
Povey et al., 2020	Y	Y	Y	L	L	★★★★
Reaven et al., 2020	Y	Y	L	Y	Y	★★★★☆
Saulsberry et al., 2013	L	Y	L	N	N	★★
Shelton et al., 2005	L	Y	Y	L	Y	★★★★
Singh et al., 2021	L	Y	L	Y	Y	★★★★
Stein et al., 2002	Y	L	Y	Y	Y	★★★★☆

*Note:* GRIPP 2 (short form) checklist (Staniszewska et al. [34]) was used.

Abbreviations: L = limited information, ☆, N = no, no star, Y = yes, ★.

### 3.3 RQ1: What Codesign Approaches Have Been Used to Develop Mental Health Interventions With Young People From Racially Minoritised Populations?

Table 4 summarises the various codesign and participatory frameworks used in the included studies. These included community‐based participatory research (*n *= 4; 22%), co‐production (*n *= 1; 6%), human‐centred design (*n *= 1; 6%), youth and family codesign model (*n *= 1; 6%), community engagement research (*n *= 1; 6%), community development model (*n *= 1; 6%), participatory evaluation model (*n *= 1; 6%), participatory research design approach (*n *= 1; 6%) and community participatory research partnership (*n *= 1; 6%). Six studies (33%) did not overtly state or indicate any specific participatory/codesign framework.

**Table 4:** Characteristics of codesign approaches.

Lead author, year	Reported codesign approach/framework used to guide study	Codesign participants	Stage(s) of development in which codesign approaches were utilised						Codesign activities reported in the study
			Initial team formation	Exploring the problem and solution	Ideating and creating	Refining	Implementing	Evaluating	
Afifi et al., 2011	Community‐based participatory research	Young people, carers and family members, and other community members (members of NGOs, funders of projects, representatives of UNRWA, community residents, members of the urban health, youth working group from the American University of Beirut)	**√**	**√**	**√**	**√**	**√**	**√**	Survey, focus group discussion, short plays, ranking and mapping
Bond et al., 2007	NR	Community members (teaching staff, site managers)	**√**	**√**	**√**	x	x	x	Interview, mapping
Brown et al., 2020	NR	Young people, caregivers, practitioners and other community members (community centre director, education officer and football academy leader)	x	**√**	**√**	**√**	**√**	**√**	Free listing interviews, semi‐structured interviews, focus group discussions, cognitive interviewing, psychologist read‐through, mock sessions (role play), adaptation workshops, structured observation and informal qualitative feedback
Clarke et al., 2022	Community‐based participatory research	Young people, other community members (community leaders, programme directors, staff and teachers)	x	**√**	**√**	**√**	x	**√**	Interviews, focus group discussions
El Guenuni et al., 2022	Co‐production	Young people, carers and family members, and other community members (members of service organisations)	**√**	**√**	**√**	**√**	**√**	**√**	Consultation, focus group discussions
Etter et al., 2019	NR	Young people, practitioners and other community member (local Inuit community)	**√**	**√**	**√**	x	x	x	Community mapping, discussion
Fabian et al., 2023	Human‐centred design approach	Young people	x	**√**	x	**√**	x	x	Think‐aloud technique
Henderson et al., 2023	Youth and family codesign model	Young people, carer and family members, and practitioners	**√**	x	**√**	x	x	**√**	Meetings
Kataoka et al., 2006	Community Participatory Research partnership	Young people, carers and family members, practitioners and other community members (teachers)	**√**	**√**	**√**	**√**	**√**	x	Meetings, focus group discussions and surveys
King and Said, 2019	NR	Young people	x	**√**	**√**	x	x	**√**	Interview
Mance et al., 2010	Community‐based participatory research,	Young people, practitioners and other community members (staff and representatives from health centre, neighbourhood residents and representatives from local youth serving agencies)	**√**	**√**	**√**	**√**	x	x	Meetings, consultation and focus group discussions
Mulvaney‐Day et al., 2006	Community‐based participatory research	Practitioners, other community members (director, administrators, teachers and literacy specialists)	**√**	**√**	**√**	**√**	x	x	Meetings, interviews, focus group discussion, workshops (critical thinking) and mapping
Povey et al., 2020	Participatory design research approaches	Young people, practitioners	**√**	**√**	**√**	**√**	x	x	Workshops (photovoice, body mapping), peer‐supported online survey
Reaven et al., 2020	Community engagement research	Carer and family members, practitioners and other community members (district officials, school personnel and self‐advocate)	x	**√**	**√**	x	x	x	Focus group discussion
Saulsberry et al., 2013	NR	Young people, carer and family members, practitioners and other community members (local community member, social worker)	x	**√**	**√**	**√**	x	x	Focus group discussion
Shelton et al., 2005	Community development model	Young people, carer and family members, and other community members (community network members)	x	**√**	**√**	x	x	x	Focus group discussion, survey and meetings
Singh et al., 2021	NR	Young people, carer and family members, practitioners and other community members (teachers, community leaders)	x	**√**	**√**	**√**	x	x	Free listing, cognitive interviews, key informant interviews and adaptation workshops
Stein et al., 2002	Participatory research partnership, Participatory evaluation methodology	Carer and family members, practitioners and other community members (school principals, teachers and programme staff)	**√**	**√**	**√**	x	**√**	**√**	NR

Abbreviations: NGO = non‐governmental organisation, NR = not reported, UNRWA = the United Nations Refugee and Works Agency.

### 3.4 RQ2: At What Phase(s) of the Developmental Process Have Codesign Approaches Been Used, and How Does Codesign Influence the Outputs of Each Phase?

As shown in Table 4, stakeholders were more frequently involved in the initial phases than in the later codesign phases. Ten studies (56%) engaged stakeholders in the ‘Initial team formation’ phase, aiming at strengthening engagement and collaboration with stakeholders. During the ‘Exploring the problem and solution’ phase, seventeen studies (94%) reported stakeholder engagement to identify community needs, and priorities. The ‘Ideating and creating’ phase was reported in seventeen studies (94%), ensuring that the intervention was codesigned with stakeholders, targeting their needs and preferences. Eleven studies (61%) highlighted stakeholders' role in the ‘Refining’ phase, reviewing and modifying interventions. The ‘Implementing’ phase involved stakeholders in five studies (28%), adapting interventions for real‐world use. Finally, in the ‘Evaluating’ phase, seven studies (39%) reported further modifications based on stakeholder feedback.

The most common data collection method was focus group discussion (*n* = 10; 56%), followed by individual interviews (*n* = 6; 33%), cognitive interviews (*n* = 2; 11%), think‐aloud technique (*n* = 1; 6%), mapping (*n* = 4; 22%), codesign workshops (*n* = 4; 22%), qualitative surveys (*n* = 4; 22%), consultations (not otherwise specified) (*n* = 2; 11%), meetings (*n* = 5; 28%), mock sessions (role play) (*n* = 1; 6%), free listing (*n* = 2; 11%) and ranking (*n* = 1; 6%). One study did not specify any data collection methods [37]. Supporting Information S3 provides further details on the different data collection methods implemented across different codesign phases.

### 3.5 RQ3: What Are the Outcomes of Codesign for the Individual Participants?

**Figure 3:**
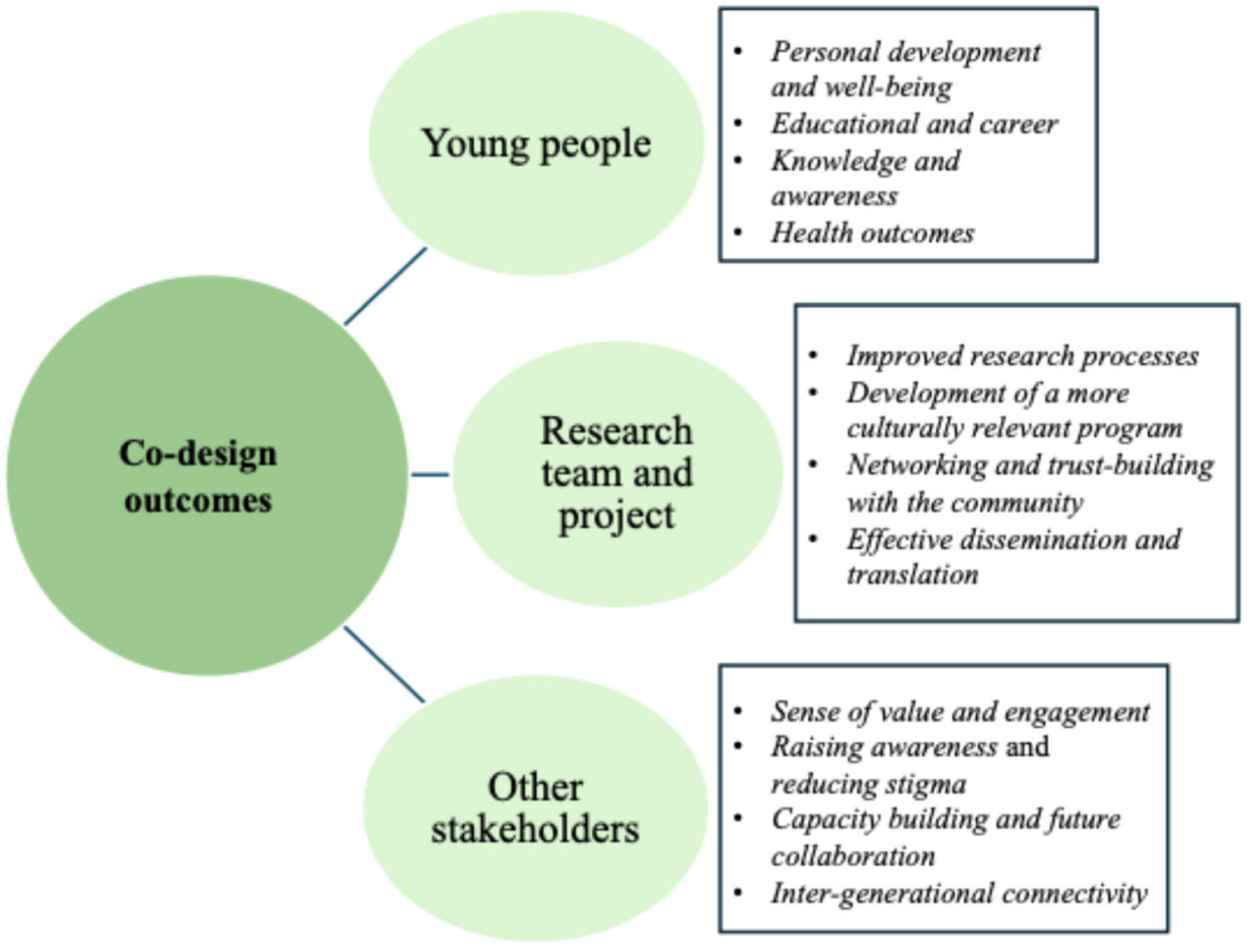
Codesign outcomes reported in the studies.

Twelve outcomes were identified and grouped into three broad categories with sub‐themes: benefits to (1) the young people, (2) the research team and project and (3) the other stakeholders (see Figure 3).

#### 3.5.1 Benefits to the Young People

Seven studies (39%) reported that youth learned to take responsibility and undertake leadership roles, which empowered them to support their peers and enhanced their self‐confidence and self‐efficacy, contributing to their personal development and well‐being [38, 39, 40, 41, 42, 43, 44]. Furthermore, five studies (28%) highlighted that youth improved their educational and career skills and enhanced their employment opportunities in health‐related fields [39, 40, 42, 43, 44]. Mance et al. [42] reported that youth were recruited as full‐time staff to serve as peer leaders for the project and were paid for their time on the project as consultants. Five studies (28%) reported that participation increased young people's knowledge and awareness of mental health issues [39, 40, 42, 44, 45], which they used to raise community awareness and encourage support‐seeking through workshops, talks and documentaries [39]. Additionally, three studies (17%) reported improved mental health outcomes among youth, as they became aware of mental health issues, learned problem‐solving skills and adopted healthy behaviours [38, 39, 44]. Out of eight studies, only three [38, 39, 41] reported outcomes based on youth feedback gathered through focus group discussions and interviews with the remaining studies, outcomes relying on the authors' reflections.

#### 3.5.2 Benefits to the Research Team and Project

Researchers in eleven studies (61%) noted that involving youth and non‐academic stakeholders improved the research process by identifying specific problems, increasing participant recruitment, enhancing data collection, gaining insights about participant experiences and developing successful grant proposals [35, 38, 39, 42, 44, 45, 46, 47, 48, 49, 50]. For instance, in one study, stakeholders from the school district offered input and direction on choosing evaluation questions and hypotheses, setting performance standards and determining data collection methods [37]. Sixteen studies (89%) reported that the codesign process enabled researchers to develop interventions that were better tailored to the cultural aspects and specific needs of the target populations [37, 38, 40, 41, 42, 43, 44, 45, 46, 48, 50, 51, 52, 53, 54]. Authors of four studies (22%) observed that codesign built trust, strengthened community partnerships and facilitated community ownership, which expanded local participant reach, provided logistical support and ensured programme sustainability [38, 42, 44, 50]. Furthermore, based on researchers' reflections, three studies (17%) reported that codesign with service users led to wider and more effective research dissemination [39, 49, 50].

#### 3.5.3 Benefits to the Other Stakeholders

In three studies (17%), it was observed that involving other stakeholders (e.g., family members, other caregivers, community members and practitioners) in codesign fostered a sense of value and engagement by creating opportunities for sharing their expertise, resources and power with the research team [38, 39, 40]. Two studies (11%) highlighted that stakeholder involvement deepened stakeholders' understanding of the mental health needs of youth and protective factors that reduced stigma and improved their access to services [39, 40]. Four studies (22%) reported that stakeholders from overburdened or underserved agencies benefited from relevant training provided through the codesign process that strengthened capacity‐building efforts within these agencies, with some staff continuing to collaborate with the research team on future projects [37, 38, 39, 47]. Additionally, two studies (11%) revealed that involving parents and older community members alongside youth fostered inter‐generational connectivity and strengthened parent–child bonds [44, 45].

### 3.6 RQ4: What Contextual Enablers and Barriers Influence the Process and Outcomes of Codesign for Different Stakeholder Groups?

Based on the meta‐synthesis of the contextual influences derived from eight papers, we identified six overarching themes of enablers and barriers. Table 5 presents each theme, sub‐theme and key examples of items within each theme.

**Table 5:** Themes and sub‐themes of reported factors that influenced the codesign process and outcomes.

Enablers		
Theme	Sub‐theme	Example
Reducing power differentials (Afifi et al., 2011; El Guenuni et al., 2022; Povey et al., 2020)	Prioritising young people's needs Positioning youth as experts Supporting youth to create their own committee Encouraging the youth to choose their group topic Including youth disengaged from school to cover a wide range of participants	‘In communities that are patriarchal, cultural norms may prevent youth from speaking vocally in front of adults, especially when they disagree. This is especially true in close‐knit small communities where everyone knows everyone else. In this case, the youth created their own committee where they felt free to talk and empowered two representatives to attend the CYC meetings.’ ‘We also worked towards trying to address the power imbalance by encouraging the young people to choose the group topics, positioning the young people as the experts, and then adapting the stabilization strategies that they were experts in; we tailored resources accordingly.’
Community engagement and building trust (Afifi et al., 2011; El Guenuni et al., 2022; Mance et al., 2010; Stein et al., 2002)	Familiarity of the research team with the community context Logistic support from local community members Attending community events and youth opportunity programmes frequently Focusing on building trusting relationships Constant communication	‘Cultural barriers were overcome through the familiarity of the research team with the community context, supported with further logistic assistance from the local committee members.’ ‘Employing local people from the community [as facilitator] who are impacted by a shared tragedy may help with community engagement and the building of trusting relationships.’ ‘We also managed their lack of trust by having a longer period of engagement and focusing on building trusting relationships.’ ‘Additionally, research team members attended community events and made frequent visits to the YO programme in an effort to strengthen trust through the development of personal relationships and proving commitment to the community.’
Collaboration and support from other stakeholders (Afifi et al., 2011; Mulvaney‐Day et al., 2006; Shelton et al., 2005)	Involving the academic team Involving organisational consultants as mediators to resolve conflicts Academic partner assistance in grant submission Commitment of stakeholders to the project	‘The presence of the academic team as a neutral party and the continuous efforts to be (and be perceived as) non‐biased facilitated the smoothing of relations.’ ‘With the assistance of their academic partners, the group submitted a grant.’ ‘When conflicts occurred, the CBPR participants worked to understand and resolve the issue, often with the organizational consultant as mediator.’

Barriers		
Theme	Sub‐theme	Example
Resource constraints (Afifi et al., 2011; Shelton et al., 2005; Singh et al., 2021; Stein et al., 2002)	Lack of funding Time limitation Lack of staff	‘The process of developing this community‐based program was interrupted numerous times due to changes in the financing of children's mental health services in Maryland and growing state budget constraints. With the tenuous nature of state funding, it became evident to the network that external funding would be required.’ ‘Consistent communication with incentive staff was challenging because mobile phones could not be provided to incentive staff and limited in‐camp transportation decreased the geographical reach.’ ‘From the perspective of academics, perhaps one of the most difficult aspects of engaging communities in planning is the time commitment required to do so. This could be viewed as a limitation of the method.’ ‘Although the NGOs were willing and ready to be engaged in the process, they found various aspects of it difficult, which resulted in each stage taking more time than originally planned.’ ‘While our approach has been aimed at achieving maximum partnership with the community by employing incentive workers with diverse backgrounds (as advised by participants during the RQA), we encountered challenges around capacity and retention of short‐term staff, and national restrictions on refugees in paid employment.’
Relationship conflicts and power dynamics (Afifi et al., 2011; El Guenuni et al., 2022; Mance et al., 2010; Mulvaney‐Day et al., 2006)	Power imbalance Lack of empowerment Cultural barriers Insufficient rapport building and trust Interpersonal dynamics Lack of mutual expectation and commitment	‘There was a power imbalance because we were adults, professionals, held positions of power, and some of us worked in the NHS; they also experienced lots of subjugation from authorities, the community and systems around them. In addition, there were difficult dynamics between bereaved and survivors in the community which were mirrored in the group space.’ ‘Networking and trust building to expand community involvement can be a challenge in any collaborative process. Rapport building and trust were particularly difficult.’ ‘Several interpersonal dynamics affected the successful implementation of the interventions: a tendency for each constituency to blame the other for the problem, a reluctance to acknowledge that current patterns of operation are not really working, and differing levels of readiness for change across participants.’ ‘Although peer leaders provided valuable lived experiences and knowledge to the partnership, establishing mutual role expectations and commitment proved challenging.’
Selection bias (Povey et al., 2020)	Selection bias	‘One of the challenges of effective co‐design is selection bias, with the inclusion of youth in the co‐design process who are engaged and generally functioning well in their school and community.’

Abbreviations: CBPR = community‐based participatory research, CYC = Community Youth Committee, NGO = non‐governmental organisation, NHS = National Health Service, RQA = rapid qualitative assessment, YO = youth opportunity.

#### 3.6.1 Enablers

Three overarching themes—reducing power differentials, community engagement and building trust, and stakeholder collaboration and support—were identified as enablers in conducting codesign research.

##### 3.6.1.1 Reducing Power Differentials

In three studies, enablers of codesign included reducing power differentials between researchers and young people. Supporting youth in taking responsibilities (e.g., planning sessions, leading discussions, presenting findings and making decisions) fostered ownership and comfort [38, 39]. Efforts to include diverse participants (age, socioeconomic status, geographical locations and language) mitigated misrepresentation of youth needs and desires [43].

##### 3.6.1.2 Community Engagement and Building Trust

Another significant enabler was community engagement and building trust, as mentioned in four studies. Attending community events and youth programmes frequently helped researchers overcome cultural barriers, build trust and foster cultural relevance [39, 42]. Moreover, the authors noted that constant communication with stakeholders and the local community strengthened collaboration, integrated cultural perspectives into the codesign process and enhanced intervention effectiveness [37, 38].

##### 3.6.1.3 Collaboration and Support From Other Stakeholders

This theme, reported in three studies, describes the effectiveness of codesign when supported by stakeholders' collaboration. Community and healthcare experts' involvement and academic partnerships facilitated engagement, resolved conflicts and developed smoother relationships [38, 47]. Additionally, academic partnerships helped secure a grant for one project [44].

#### 3.6.2 Barriers

Three main barriers to meaningful codesign were resource constraints, relationship conflicts and power dynamics, and selection bias.

##### 3.6.2.1 Resource Constraints

The most common barrier to codesign, reported in four studies involved resource limitations. Budget constraints, lack of research grants and absence of incentives for participation hindered the codesign process [44, 49]. Time limitations were another significant challenge, as engaging communities in the codesign required substantive time investment with each stage often taking longer than originally planned [37, 38]. Additionally, there was a lack of staff to effectively support the codesign process [49].

##### 3.6.2.2 Relationship Conflicts and Power Dynamics

Relationship conflicts and power dynamics between researchers and stakeholders was another barrier, mentioned in four studies. Working with adolescents and youth often created power imbalances due to the researchers' authoritative positions [39]. Moreover, networking and trust building to expand community engagement was another challenge [42]. Interpersonal dynamics and relationship conflicts affected the successful implementation of interventions [47]. Cultural norms in some communities, particularly patriarchal ones, also prevented youth involvement in the research [38].

##### 3.6.2.3 Selection Bias

Selection bias was identified in one study as a barrier to effective codesign. This study included only youth who were already engaged and functioning well in school and community and potentially misrepresents the needs of disengaged youth, who might benefit most from mental health interventions [43].

## 4 Discussion

This review comprehensively examined codesign approaches in developing mental health interventions for racially minoritised young people. Eighteen studies were identified involving youth and other stakeholders, primarily in early codesign stages using various data collection methods. codesign participation enhanced young people's knowledge, leadership skills and career opportunities. It enabled researchers to develop culturally aligned interventions by exploring youth's lived experiences while ensuring that all stakeholders felt valued through sharing their expertise, power and resources. Although resource limitations, power imbalances and sampling issues were noted as challenges, the process was facilitated by reducing power differentials, encouraging community engagement and stakeholders' collaboration.

The review identified diverse codesign models, highlighting the flexibility of participatory methodologies. Although focused on youth, other stakeholders were engaged to better understand minoritised communities' interconnectedness. Therefore, most studies prioritised community partnership as connections among individuals, families and communities shape community perceptions [22]. Previous research shows that the explanatory models of community influence codesign research structure and service development [55, 56, 57].

Our findings indicated a trend of early‐stage involvement with limited participation in later stages, consistent with previous research [58] and noted various data collection activities across these phases. Previous studies suggest that early involvement empowers young people to shape research direction, amplifying their sense of impact on outcomes [59, 60], whereas another study indicated that involvement in every stage may not be necessary for meaningful contribution [61]. However, our findings of relatively limited involvement in later stages of codesign also raise questions about tokenism, where youth are given initial expertise but lack real influence over the process and outcomes [62]. This issue, compounded by researchers' authoritative role, reinforces power imbalances and reflects epistemic injustice in mental health research, where the lived experiences of racially minoritised youth are undervalued compared to ‘professional’ knowledge [63].

The studies in this review reported using various codesign activities. Research suggests that the diversity of methods and activities used in codesign is a key strength, as it enables researchers to tailor involvement to the specific needs of racially minoritised youth [58]. Flexible and adaptive participation addresses practical challenges such as low literacy rates, cultural biases and confidentiality concerns. From an epistemic justice perspective, accommodating diverse ways of expressing and validating the lived experiences of racially minoritised youth can create an inclusive environment that respects and amplifies the unique insights these youth bring to the research process [63]. However, details on the adaptation of these activities were often lacking in included studies, with no insights on which activities were most effective and acceptable for these youth.

Building on previous findings [64, 65], this review highlights the outcomes of codesign for young people, research projects and other stakeholders. However, a significant gap exists in the literature as it focuses primarily on the general population, often overlooking the distinct experiences and needs of racially minoritised communities. These communities face unique challenges within the mental health system, where traditional Western models of care may not align with their cultural understandings of mental health or adequately address systemic biases that impact service delivery [7, 8, 10, 11]. As a result, their mental health needs often go unmet, contributing to disparities in access, engagement and outcomes [2, 3]. Codesign offers a way to address these deficits by involving racially minoritised youth as active contributors to research and intervention design, ensuring that mental health interventions are not only culturally and contextually relevant but also responsive to the lived experiences of those most affected by systemic barriers [23]. The studies in this review demonstrate that codesign connected youth, researchers and other community stakeholders, with participants benefiting directly or indirectly. By adopting codesign approaches, researchers developed culturally and contextually relevant interventions grounded in the lived experiences of target populations. This approach also ensured that youth and other stakeholders' expertise were heard and valued, leading to mutual benefits and improved research outcomes [42]. However, only a few studies directly included young people's feedback on intervention outcomes with the majority relying on researcher observations. This may limit the comprehensiveness of the evaluation and potentially overlook important insights from the youth themselves.

This review identified significant barriers and enablers in codesign studies, consistent with previous reviews on codesign with young people [65, 66]. It is crucial to support participants, particularly racially minoritised youth who may experience social stigma and power imbalances within certain community and societal contexts. Addressing these imbalances is essential for effective codesign, as unequal power dynamics can undermine trust and lead to negative outcomes [67, 68].

### 4.1 Strengths and Limitations

Despite the restricted evidence base and varying degrees of stakeholder involvement in the reviewed studies, our findings offer insights into useful codesign approaches with young people and their families. This review combines diverse evidence from qualitative and mixed‐method studies, providing comprehensive insights. However, the review has some limitations. It is constrained by the availability of published English peer‐reviewed literature, potentially missing relevant studies. The lack of robust methodological descriptions in the included studies makes it difficult to assess the comparative effectiveness of codesign approaches. Moreover, most included studies are from high‐income countries, limiting the generalisation of our findings to low‐ and middle‐income countries. We did not categorise the impact of codesign on youth by their developmental age because the small number of included studies made it difficult to provide meaningful insights based on age categories. One theme in the synthesis on barriers was supported by only one study, highlighting the need for further research on the prevalence and impacts of selection bias in codesign processes. Finally, we were unable to involve youth consultants in our review due to resource constraints, which might have enhanced the relevance of our findings.

### 4.2 Recommendations for Codesigning With Racially Minoritised Young People

Effective codesign with racially minoritised young people, as with any specific population, requires adapting approaches to meet their unique needs and challenges, which may include addressing intersectional issues such as systemic racism, cultural stigma and community‐specific barriers to participation. In light of this, and considering our findings, we make the following recommendations:

1. Codesign methodologies should be contextually sensitive, integrating the lived experiences, values and needs of racially minoritised communities.
2. Given the history of past exploitation and misrepresentation of racially minoritised populations in research, codesign should prioritise building long‐term relationships based on transparency, respect and reciprocity while actively addressing power imbalances by giving youth meaningful decision‐making roles.
3. Researchers should consider offering incentives like travel expenses or stipends to address financial barriers that may limit youth participation.
4. Long‐term engagement strategies such as ongoing support, skill development and vocational pathways can help young individuals to influence research and policy beyond the life span of the project. Building community capacity helps to ensure that codesign benefits are sustained.
5. To enhance evaluation in future codesign research, objective measurements such as tracking involvement activities, assessing participant feedback and gauging satisfaction levels should be employed. Combining these quantitative measures with qualitative feedback directly from youth can provide a more holistic evaluation.

## 5 Conclusion

This review highlights the value of codesign in developing culturally relevant mental health interventions for racially minoritised youth. Our findings suggest that continuous engagement with these young people and all stakeholders throughout the codesign process will help to address barriers such as resource limitations, power dynamics and cultural differences. Acknowledging these communities' strengths and unique needs can strengthen trust, balance power and lead to a more effective and inclusive codesign process. Additionally, integrating community‐specific frameworks and combining qualitative and quantitative evaluation methods will help in developing effective and culturally relevant interventions.

## Author Contributions

**Ishrat Shahnaz:** conceptualization, writing – original draft, data curation, methodology, investigation, formal analysis, project administration, software. **Kelly Rose‐Clarke:** conceptualization, methodology, validation, supervision, writing – review and editing. **Daniel Michelson:** conceptualization, validation, writing – review and editing, supervision, methodology. **Petra C. Gronholm:** conceptualization, methodology, validation, writing – review and editing, supervision.

## Conflicts of Interest

The authors declare no conflicts of interest.

## Supporting information

Supporting information.

Supporting information.

Supporting information.

## Data Availability

The authors have nothing to report.
