# Supplementary material for: Codesign of Mental Health Interventions With Young People From Racially Minoritised Populations: A Systematic Review of Methods and Outcomes
Source: Health Expect. 2025 Mar 6;28(2):e70204. doi: 10.1111/hex.70204 (PMC11883660; doi:10.1111/hex.70204)
Supplement: Supplementary file 2 — Supporting information. [file HEX-28-e70204-s002.docx]

**Full Search Strategy**

**Ovid MEDLINE(R) ALL <1946 to 2023>**

**Search run 21/07/2023**

| **#** |  | **Results** |
| --- | --- | --- |
| **1** | exp Community-Based Participatory Research/ | **6015** |
| **2** | *Patient Participation/ or *Community Participation/ or *Stakeholder Participation/ or *Community Involvement/ | **27673** |
| **3** | (collaborat* or consult* or participat* or partner* or co-develop* or codevelop* or co develop* or co-design* or co design* or codesign* or coproduc* or co-produc* or co produc* or adapt* or cocreat* or co-creat* or co creat*).tw. | **1829556** |
| **4** | Or/1-3 | **1846565** |
| **5** | *Mental Health/ or *Mental Disorders/ | **169979** |
| **6** | ((mental health* or psychiatr* or psycho* or emotion*) adj2 (illness* or condition* or disorder* or impair* or difficult* or problem* or symptom*)).tw. | **220208** |
| **7** | *Mood Disorders/ or *Depression/ | **96789** |
| **8** | (affective disorder* or bipolar* or mania or manic or cyclothymic* or depress* or dysthymi* or MDD).tw. | **625204** |
| **9** | *Anxiety Disorders/ or *Stress Disorders, Traumatic, Acute/ | **25597** |
| **10** | (agoraphobi* or anxi* or GAD or phobi* or panic* or obsess* or compulsi* or OCD or post$trauma* or stress* or distress* or PTSD or trauma*).tw. | **1840090** |
| **11** | *Child Behavior Disorders/ or *Conduct Disorders/ or *Aggression/ | **37515** |
| **12** | ((antisocial or anti-social or disruptive or conduct* or oppositional or behavio*) adj2 (behavio?r or problem* or difficult* or disorder* or symptom*)).tw. | **1051201** |
| **13** | Or/5-12 | **3376884** |
| **14** | *Psychosocial Intervention/ or *Psychotherapy/ or *Counselling/ or *Behaviour Therapy/ or *Treatment/ | **66114** |
| **15** | (intervention* or program* or therap* or counsel* or prevent* or promot* or workshop* or support* or service* or training*).tw. | **9177893** |
| **16** | Or/14-15 | **9202422** |
| **17** | *Adolescents/ or *Child/ | **8575** |
| **18** | (child* or teen* or youth* or adolescen* or juvenile* or (young adj2 (adult* or person* or people*)) or school-age* or ((secondary or high*) adj2 (school* or education))).tw. | **2092214** |
| **19** | Or/17-18 | **2094110** |
| **20** | *"Minority Group"/ Or *"Black Person"/ Or *"Asian Continental Ancestry Group"/ Or *"British Asian"/ Or *"Ethnic Group"/ Or *"Black Race"/ Or *"Black Population"/ Or *"Multiracial Person"/ Or *"Indigenous People"/ Or *"Asian American"/ Or *African American/ Or *Ethnicity/ Or *African Continental Ancestry Group/ Or *American Native Continental Ancestry Group/ | **120998** |
| **21** | (race* or minorit* or racial or multi ethnic* or multi?ethnic* or multi?racial* or multi?lingual or multi lingual or multi?cultural or multi cultural or divers* or diverse population* or cultural diversit* or racial disparit*).tw. | **990885** |
| **22** | *"Transients And Migrants"/ Or *"Emigrants And Immigrants"/ Or *Refugees/ | **31891** |
| **23** | (migrant* or immigrant* or emigrant* or refugee* or asylum seek*).tw. | **63340** |
| **24** | ("BAME" or BME or african* or "afro$caribbean" or caribbean or asia* or bangladesh* or nepal* or india* or chin* or pakistan* or sri lanka* or taiwan* or vietnam* or korea* or japan or malaysia* or philipin* or thai* or combodia* or indonesi* or arab* or gyps* or irish traveller* or roma or jew* or hispanic*).tw. | **1705713** |
| **25** | Or/20-24 | **2618308** |
| **26** | 4 and 13 and 16 and 19 and 25 | **8741** |
|  | Limit 26 to (English language) | **8491** |
|  | After Deduplication | **8465** |

**Embase via Ovid <1974 to 2023>**

**Search run 21/07/2023**

| **#** |  | **Results** |
| --- | --- | --- |
| **1** | *community-based participatory research/ or *Patient Participation/ or *Community Participation/ or *Stakeholder Participation/ | **14332** |
| **2** | (collaborat* or consult* or participat* or partner* or co-design* or co design* or codesign* or coproduc* or co-produc* or co produc* or adapt* or cocreat* or co-creat* or co creat*).tw. | **2397276** |
| **3** | Or/1-3 | **2403997** |
| **4** | *Mental Health/ or *Mental Disease/ | **169109** |
| **5** | ((mental health* or psychiatr* or psycho* or emotion*) adj2 (illness* or condition* or disorder* or impair* or difficult* or problem* or symptom*)).tw. | **303811** |
| **6** | *Mood Disorders/ or *Depression/ | **190348** |
| **7** | (affective disorder* or bipolar* or mania or manic or cyclothymic* or depress* or dysthymi* or MDD).tw. | **839603** |
| **8** | *Anxiety Disorders/ or *Stress Disorders, Traumatic, Acute/ | **11563** |
| **9** | (agoraphobi* or anxi* or GAD or phobi* or panic* or obsess* or compulsi* or OCD or post$trauma* or stress* or distress* or PTSD or trauma*).tw. | **2348653** |
| **10** | *Child Behavior Disorders/ or *Conduct Disorder/ or *Aggression/ | **37751** |
| **11** | ((antisocial or anti-social or disruptive or conduct* or oppositional or behavio*) adj2 (behavio?r or problem* or difficult* or disorder* or symptom*)).tw. | **1183206** |
| **12** | Or/4-11 | **4115429** |
| **13** | *Psychosocial Intervention/ or *Psychotherapy/ or *Counselling/ or *Behaviour Therapy/ | **75863** |
| **14** | (intervention* or program* or therap* or counsel* or prevent* or promot* or workshop* or support* or service* or training*).tw. | **12074100** |
| **15** | Or/13-14 | **12097145** |
| **16** | *Adolescent/ or *Child/ | **103933** |
| **17** | (child* or teen* or youth* or adolescen* or juvenile* or (young adj2 (adult* or person* or people*)) or school-age* or ((secondary or high*) adj2 (school* or education))).tw. | **2640435** |
| **18** | Or/16-17 | **2649014** |
| **19** | *"Minority Group"/ Or *"Black Person"/ Or *"Asian Continental Ancestry Group"/ Or *"British Asian"/ Or *"Ethnic Group"/ Or *"Black Race"/ Or *"Black Population"/ Or *"Multiracial Person"/ Or *"Indigenous People"/ Or *"Asian American"/ Or *African American/ Or *Ethnicity/ Or *African Continental Ancestry Group/ Or *American Native Continental Ancestry Group/ | **69142** |
| **20** | (race* or minorit* or racial or multi ethnic* or multi?ethnic* or multi?racial* or multi?lingual or multi lingual or multi?cultural or multi cultural or divers* or diverse population* or cultural diversit* or racial disparit*).tw. | **1205485** |
| **21** | *"Transients And Migrants"/ Or *"Emigrants And Immigrants"/ Or *Refugees/ | **33411** |
| **22** | (migrant* or immigrant* or emigrant* or refugee* or asylum seek*).tw. | **70230** |
| **23** | ("BAME" or BME or african* or "afro$caribbean" or caribbean or asia* or bangladesh* or nepal* or india* or chin* or pakistan* or sri lanka* or taiwan* or vietnam* or korea* or japan or malaysia* or philipin* or thai* or combodia* or indonesi* or arab* or gyps* or irish traveller* or roma or jew* or hispanic*).tw. | **2198372** |
| **24** | Or/19-23 | **3271134** |
| **25** | 4 and 13 and 16 and 19 and 25 | **11561** |
|  | limit 25 to (English language) | **11269** |
|  | After deduplication | **11121** |

**PsycINFO (via Ovid) <1806 to 2023>**

**Search run 16/07/2023**

| **#** |  | **Results** |
| --- | --- | --- |
| **1** | *Patient Participation/ or *Community Participation/ or *Stakeholder Participation/ or *Community Involvement/ | **7242** |
| **2** | (collaborat* or consult* or participat* or partner* or co-develop* or codevelop* or co develop* or "co-design" or "co design" or "codesign" or coproduc* or co-produc* or co produc* or adapt* or cocreat* or co-creat* or co creat*).tw. | **750434** |
| **3** | Or/1-2 | **752961** |
| **4** | *Mental Health/ | **65427** |
| **5** | ((mental health* or psychiatr* or psycho* or emotion*) adj2 (illness* or condition* or disorder* or impair* or difficult* or problem* or symptom*)).tw. | **225194** |
| **6** | *Mood Disorders/ | **12040** |
| **7** | (affective disorder* or bipolar* or mania or manic or cyclothymic* or depress* or dysthymi* or internali* or MDD).tw. | **425108** |
| **8** | *Anxiety Disorders/ or *Acute stress disorder/ or *Posttraumatic stress disorder | **50463** |
| **9** | (agoraphobi* or anxi* or GAD or phobi* or panic* or obsess* or compulsi* or OCD or post$trauma* or stress* or distress* or PTSD or trauma*).tw. | **690102** |
| **10** | *Behavior Disorders/ or *Conduct Disorder/ or *Aggressive Behavior/ | **34397** |
| **11** | ((antisocial or anti-social or disruptive or conduct* or oppositional or behavio*) adj2 (behavio?r or problem* or difficult* or disorder* or symptom*)).tw. | **772712** |
| **12** | Or/4-11 | **1684219** |
| **13** | *Psychotherapy/ or *Counseling/ or *Behavior Therapy/ | **82310** |
| **14** | (intervention* or program* or therap* or counsel* or prevent* or promot* or workshop* or support* or service* or training*).tw. | **2253596** |
| **15** | Or/13-14 | **2266202** |
| **16** | *Students/ | **15924** |
| **17** | (child* or teen* or youth* or adolescen* or juvenile* or (young adj2 (adult* or person* or people*)) or school-age* or ((secondary or high*) adj2 (school* or education))).tw. | **1180870** |
| **18** | Or/16-17 | **1190421** |
| **19** | *"Minority Group"/ Or *"Black Person"/ Or *"Asian Continental Ancestry Group"/ Or *"British Asian"/ Or *"Ethnic Group"/ Or *"Black Race"/ Or *"Black Population"/ Or *"Multiracial Person"/ Or *"Indigenous Population"/ Or *"Asian American"/ Or *African American/ Or *Ethnicity/ Or *African Continental Ancestry Group/ Or *American Native Continental Ancestry Group/ | **72207** |
| **20** | (race* or minorit* or racial or multi ethnic* or multi?ethnic* or multi?racial* or multi?lingual or multi lingual or multi?cultural or multi cultural or divers* or diverse population* or cultural diversit* or racial disparit*).tw. | **326280** |
| **21** | *Refugees/ | **7119** |
| **22** | (migrant* or immigrant* or emigrant* or refugee* or asylum seek*).tw. | **51805** |
| **23** | ("BAME" or BME or african* or "afro$caribbean" or caribbean or asia* or bangladesh* or nepal* or india* or chin* or pakistan* or sri lanka* or taiwan* or vietnam* or korea* or japan or malaysia* or philipin* or thai* or combodia* or indonesi* or arab* or gyps* or irish traveller* or roma or jew* or hispanic*).tw. | **349598** |
| **24** | Or/19-23 | **655701** |
| **25** | 3 and 12 and 15 and 18 and 24 | **10989** |
|  | limit 25 to (English language) | **10322** |
|  | After deduplication | **10312** |

**Global Health via Ovid <1973 to 2023>**

**Search run 16/07/2023**

| **#** |  | **Results** |
| --- | --- | --- |
| **1** | Participation/ or Community involvement/ | **11380** |
| **2** | (Patient Participation or Community Participation or Stakeholder Participation or Community-based participatory research).mp. | **4094** |
| **3** | (collaborat* or consult* or participat* or partner* or co-develop* or codevelop* or co develop* or "co-design" or "co design" or "codesign" or coproduc* or co-produc* or co produc* or adapt* or cocreat* or co-creat* or co creat*).mp. | **308969** |
| **4** | Or/1-3 | **310543** |
| **5** | Mental Health/ or Mental Disorders/ | **81850** |
| **6** | ((mental health* or psychiatr* or psycho* or emotion*) adj2 (illness* or condition* or disorder* or impair* or difficult* or problem* or symptom*)).mp. | **28774** |
| **7** | Depression/ | **34577** |
| **8** | (mood* or affective disorder* or bipolar* or mania or manic or cyclothymic* or depress* or dysthymi* or MDD).mp. | **74035** |
| **9** | Anxiety/ or Mental Stress/ | **33029** |
| **10** | (agoraphobi* or anxi* GAD or phobi* or panic* or obsess* or compulsi* or OCD or post$trauma* or stress* or distress* or PTSD or trauma*).mp. | **253958** |
| **11** | Behaviour problems/ or Behaviour disorders/ or Aggressive behaviour/ | **24503** |
| **12** | ((antisocial or anti-social or disruptive or conduct* or oppositional or behavio*) adj2 (behavio?r or problem* or difficult* or disorder* or symptom*)).mp. | **250207** |
| **13** | Or/5-12 | **541036** |
| **14** | Psychotherapy/ or Counselling/ | **14878** |
| **15** | (intervention* or program* or therap* or counsel* or prevent* or promot* or workshop* or support* or service* or training*).mp. | **1647025** |
| **16** | Or/14-15 | **1647129** |
| **17** | Adolescents/ or Children/ | **373655** |
| **18** | (child* or teen* or youth* or adolescen* or juvenile* or (young adj2 (adult* or person* or people*)) or school-age* or ((secondary or high*) adj2 (school* or education))).mp. | **528749** |
| **19** | Or/17-18 | **528749** |
| **20** | Minorities/ Or Black People/ Or Ethnic Group/ Or Indigenous People/ or African American/ Or Ethnicity/ | **83406** |
|  | ("Asian Continental Ancestry Group" or "British Asian" or "Black Race" or "Multiracial Person" or "Asian American" or "African Continental Ancestry Group" or "American Native Continental Ancestry Group").mp. | **2218** |
| **21** | (race* or minorit* or racial or multi ethnic* or multi?ethnic* or multi?racial* or multi?lingual or multi lingual or multi?cultural or multi cultural or divers* or diverse population* or cultural diversit* or racial disparit*).mp. | **190474** |
| **22** | Migrants/ Or Immigrants/ Or Refugees/ | **17099** |
| **23** | (migrant* or immigrant* or emigrant* or refugee* or asylum seek*).mp. | **26483** |
| **24** | ("BAME" or BME or african* or "afro$caribbean" or caribbean or asia* or bangladesh* or nepal* or india* or chin* or pakistan* or sri lanka* or taiwan* or vietnam* or korea* or japan or malaysia* or philipin* or thai* or combodia* or indonesi* or arab* or gyps* or irish traveller* or roma or jew* or hispanic*).mp. | **1032953** |
| **25** | Or/20-24 | **1190336** |
| **26** | 4 and 13 and 16 and 19 and 25 | **6689** |
|  | Limit 26 to (English language) | **6208** |
|  | After Deduplication | **6205** |

**Web of Science**

**Search run 17/03/2023**

| **#** |  | **Results** |
| --- | --- | --- |
| **1** | TS=(("Community-Based Participatory Research" or "Patient Participation" or "Community Participation" or "Stakeholder Participation" or "Community Involvement")) | **23,492** |
| **2** | AB=((collaborat* or consult* or participat* or partner* or co-develop* or codevelop* or co develop* or "co-design" or "co design" or "codesign" or coproduc* or co-produc* or co produc* or adapt* or cocreat* or co-creat* or co creat*)) | **3,637,314** |
| **3** | #1 OR #2 | **3,642,907** |
| **4** | AB=("Mental Health") | **200,355** |
| **5** | TS=(("mental health*" or psychiatr* or psycho* or emotion*) NEAR/2 (illness* or condition* or disorder* or impair* or difficult* or problem* or symptom*)) | **295,105** |
| **6** | TS=("mood disorder*" or "affective disorder*" or bipolar* or mania or manic or cyclothymic* or depress* or dysthymi* or MDD) | **952,101** |
| **7** | TS=((agoraphobi* or anxi* or GAD or phobi* or panic* or obsess* or compulsi* or OCD or post$trauma* or stress* or distress* or PTSD or trauma*)) | **3,509,709** |
| **8** | TS=("Child Behavior Disorders" or Aggression) | **78,645** |
| **9** | AB=(((antisocial or anti-social or disruptive or conduct* or oppositional or behavio*) NEAR/2 (behavio?r or problem* or difficult* or disorder* or symptom*))) | **816,021** |
| **10** | #4 OR #5 OR #6 OR #7 OR #8 OR #9 | **5,048,566** |
| **11** | TS=(("Psychosocial Intervention" or "Psychotherapy" or "Counselling" or "Behaviour Therapy" or "Treatment")) | **5,836,213** |
| **12** | AB=((intervention* or program* or therap* or counsel* or prevent* or promot* or workshop* or support* or service* or training*) ) | **11,829,131** |
| **13** | #11 OR #12 | **15,331,332** |
| **14** | TS=((child* or teen* or youth* or adolescen* or juvenile* or (young NEAR/2 (adult* or person* or people*)) or school-age* or ((secondary or high*) NEAR/2 (school* or education)))) | **3,230,748** |
| **15** | TS=(("Minority Group" Or "Black Person" Or "Asian Continental Ancestry Group" Or "British Asian" Or "Ethnic Group" Or "Black Population" Or "Multiracial Person" Or "Indigenous People" Or "Asian American" Or "African American" Or Ethnicity Or "African Continental Ancestry Group" Or "American Native Continental Ancestry Group")) | **244,589** |
| **16** | AB=(((race* or racial or multi ethnic* or multi?ethnic* or multi?lingual or multi lingual or multi?cultural or multi cultural or divers* or diverse population* or cultural diversit* or racial disparit*))) | **1,551,031** |
| **17** | TS=((migrant* or immigrant* or emigrant* or refugee* or asylum seek*)) | **188,729** |
| **18** | AB=(("BAME" or BME or african* or "afro$caribbean" or caribbean or asia* or bangladesh* or nepal* or india* or chin* or pakistan* or sri lanka* or taiwan* or vietnam* or korea* or japan or malaysia* or philipin* or thai* or combodia* or indonesi* or arab* or gyps* or irish traveller* or roma or jew* or hispanic*)) | **2,868,533** |
| **19** | #15 OR #16 OR #17 OR #18 | **4,416,399** |
| **20** | #3 AND #10 AND #13 AND #14 AND #19 | **11,028** |
| **21** | #3 AND #10 AND #13 AND #14 AND #19 and English (Languages) | **10,755** |
|  | After Deduplication | **10749** |

**Scopus**

**Search run 21/07/2023**

| **#** |  | **Results** |
| --- | --- | --- |
| **1** | TITLE-ABS-KEY( "community-based participatory research"  OR  "Patient Participation"  OR  "Community Participation"  OR  "Stakeholder Participation"  OR  "Community Involvement" ) | **80,410** |
| **2** | TITLE-ABS ( collaborat*  OR  consult*  OR  participat*  OR  partner*  OR  co-develop*  OR  codevelop*  OR  "co develop*"  OR  "co-design*"  OR  "co design*"  OR  "codesign*"  OR  coproduc*  OR  co-produc*  OR  "co produc*"  OR  adapt*  OR  cocreat*  OR  co-creat*  OR  "co creat*" ) | **4,879,964** |
| **3** | #1 or #2 | **4,913,990** |
| **4** | TITLE-ABS-KEY( "Mental Health"  OR  "Mental Disorders" ) | **616,845** |
| **5** | TITLE-ABS ( ( "mental health*"  OR  psychiatr*  OR  psycho*  OR  emotion* )  W/2  ( illness*  OR  condition*  OR  disorder*  OR  impair*  OR  difficult*  OR  problem*  OR  symptom* ) ) | **346,587** |
| **6** | TITLE-ABS ( "mood disorder*"  OR  "affective disorder*"  OR  bipolar*  OR  mania  OR  manic  OR  cyclothymic*  OR  depress*  OR  dysthymi*  OR  mdd ) | **967,925** |
| **7** | TITLE-ABS-KEY( "Anxiety Disorders"  OR  "Stress Disorders" ) | **208,374** |
| **8** | TITLE-ABS ( agoraphobi*  OR  anxi*  OR  gad  OR  phobi*  OR  panic*  OR  obsess*  OR  compulsi*  OR  ocd  OR  post$trauma*  OR  stress*  OR  distress*  OR  ptsd  OR  trauma* ) | **285,082** |
| **9** | TITLE-ABS-KEY( "Child Behavior Disorders"  OR  "Aggression" ) | **124,526** |
| **10** | TITLE-ABS ( ( antisocial  OR  anti-social  OR  disruptive  OR  conduct*  OR  oppositional  OR  behavio* )  W/2  ( behavio?r  OR  problem*  OR  difficult*  OR  disorder*  OR  symptom* ) ) | **1,272,989** |
| **11** | #4 or #5 or #6 or #7 or #8 or #9 or #10 | **3,140,603** |
| **12** | TITLE-ABS-KEY( "Psychosocial Intervention"  OR  "Psychotherapy"  OR  "Counselling"  OR  "Behaviour Therapy"  OR  "Treatment" ) | **10,016,572** |
| **13** | TITLE-ABS ( intervention* OR  program*  OR  therap*  OR  counsel*  OR  prevent*  OR  promot*  OR  workshop*  OR  support*  OR  service*  OR  training* ) | **18,599,052** |
| **14** | #12 or #13 | **24,467,849** |
| **15** | TITLE-ABS-KEY( child*  OR  teen*  OR  youth*  OR  adolescen*  OR  juvenile*  OR  ( ( young )  W/2  ( adult*  OR  person*  OR  people* ) )  OR  school-age*  OR  ( ( secondary  OR  high* )  W/2  ( school*  OR  education ) ) ) | **6,252,140** |
| **16** | TITLE-ABS ( "Minority Group"  OR  "Black Person"  OR  "Asian Continental Ancestry Group"  OR  "British Asian"  OR  "Ethnic Group"  OR  "Black Race"  OR  "Black Population"  OR  "Multiracial Person"  OR  "Indigenous People"  OR  "Asian American"  OR  "African American"  OR  "Ethnicity"  OR  "African Continental Ancestry Group"  OR  "American Native Continental Ancestry Group" ) | **311,431** |
| **17** | TITLE-ABS-KEY( race*  OR  minorit*  OR  racial  OR  "multi ethnic*"  OR  multi?ethnic*  OR  multi?racial*  OR  multi?lingual  OR  "multi lingual"  OR  multi?cultural  OR  "multi cultural"  OR  divers*  OR  "diverse population*"  OR  "cultural diversit*"  OR  "racial disparit*" ) | **2,525,818** |
| **18** | TITLE-ABS-KEY ( "Transients And Migrants"  OR  "Emigrants And Immigrants") | **27,273** |
| **19** | TITLE-ABS-KEY( migrant*  OR  immigrant*  OR  emigrant*  OR  refugee*  OR  "asylum seek*" ) | **242,252** |
| **20** | TITLE-ABS ( "BAME"  OR  bme  OR  african*  OR  afro$caribbean  OR  caribbean  OR  asia*  OR  bangladesh*  OR  nepal*  OR  india*  OR  chin*  OR  pakistan*  OR  "sri lanka*"  OR  taiwan*  OR  vietnam*  OR  korea*  OR  japan  OR  malaysia*  OR  philipin*  OR  thai*  OR  combodia*  OR  indonesi*  OR  arab*  OR  gyps*  OR  "irish traveller*"  OR  roma  OR  jew*  OR  hispanic* ) | **66,554** |
| **21** | #16 or #17 or #18 or #19 or #20 | **2,917,502** |
| **22** | #3 and #11 and #14 and #15 and #21 | **7,925** |
|  | Limit 22 to (English language) | **7,639** |
|  | After Deduplication | **7634** |
